# Recent Advances and Future Perspectives in Biosensors for Monitoring and Diagnostics

**DOI:** 10.3390/bios16050237

**Published:** 2026-04-23

**Authors:** Radivoje Prodanović, Dalibor Stanković

**Affiliations:** University of Belgrade-Faculty of Chemistry, Studentski Trg 12, 11000 Belgrade, Serbia

## 1. Introduction

Biosensors have become some of the most vital analytical tools in modern biotechnology, medicine, environmental monitoring, food safety, and industrial process control. They combine biological recognition elements, such as enzymes, antibodies, nucleic acids, or cells, with physical and chemical transducers, such as electrochemical, optical, piezoelectric, and electronic detection systems. These systems allow for selective detection of biologically relevant compounds and are used in medical diagnostics, environmental monitoring, food analysis, and biotechnology.

Over the past few decades, biosensor technology has evolved from simple enzyme electrodes to advanced integrated analytical systems that combine biological recognition, nanomaterials, microelectronics, microfluidics, and data processing. Modern biosensors are increasingly integrated into portable electronics and wearable devices, enabling continuous monitoring and point-of-care diagnostics.

## 2. Recent Developments in Biosensors

In recent years, biosensor research has shifted toward smaller, portable, and intelligent sensing systems capable of real-time monitoring and data analysis. Advances in biosensors have been driven by progress in nanomaterials, microfluidics, wearable electronics, molecular diagnostics, and bioinformatics. Nanomaterials such as metal nanoparticles, carbon nanomaterials, quantum dots, metal–organic frameworks, and polymer nanocomposites have enhanced the sensitivity, stability, and miniaturization of biosensors. These nanostructured materials provide a high surface area, improved electron transfer, and better immobilization of biological molecules, significantly enhancing biosensor performance.

Advances in molecular diagnostics, including clustered regularly interspaced short palindromic repeats (CRISPR)-based detection systems, nucleic acid sensors, and immunoassays, have enabled fast and multiplexed detection of biomarkers and pathogens. Microfluidic systems have supported the development of smaller analytical devices that use less sample and provide fast results. Wearable biosensors are another rapidly growing area, particularly for continuous monitoring of physiological parameters such as glucose, lactate, and oxygen saturation, as well as other biomarkers.

Artificial intelligence and data analysis tools are also becoming increasingly essential for biosensor signal processing, pattern recognition, and the creation of smart diagnostic systems.

## 3. Challenges and Knowledge Gaps

Despite notable progress, several challenges persist in the field of biosensors. These include maintaining the long-term stability of biological recognition elements, ensuring reproducibility during biosensor fabrication, integrating biosensors with electronic systems, enabling real-time data processing, and transforming laboratory biosensor systems into practical and commercial devices.

Another key challenge is developing biosensors for early disease detection, monitoring environmental pollutants at low levels, and creating continuous monitoring systems for personalized medicine. Developing new materials, improved immobilization techniques, and sophisticated signal-processing methods will be essential for addressing these challenges.

Besides technological challenges, there is also a gap between biosensor research in labs and real-world use. Many biosensor systems show excellent performance in controlled lab conditions but face issues with stability, reproducibility, cost, and large-scale manufacturing. Additionally, integrating biosensors with data processing, wireless communication, and artificial intelligence is an important area for future research. Therefore, interdisciplinary approaches that combine materials science, biotechnology, electronics, and data science are crucial for developing next-generation biosensor systems.

An important research area and knowledge gap concerns the design and engineering of biological recognition elements for biosensors. While enzymes, antibodies, and nucleic acids are commonly used as sensing elements, their stability, selectivity, and electron-transfer properties are often not ideal for biosensor design. Protein engineering, directed evolution, and synthetic biology provide powerful tools for creating biological components with better stability, catalytic activity, and electron-transfer efficiency. However, combining protein engineering with biosensor design, materials science, and electronic systems is still a developing field and an important direction for future biosensor advancements.

Addressing these challenges will require strong interdisciplinary collaboration between chemists, biochemists, materials scientists, engineers, and data scientists.

## 4. Overview of the Special Issue

This Special Issue, titled “Biosensors for Monitoring and Diagnostics”, contains research and review articles exploring key areas of biosensor research, including molecular diagnostics, electrochemical and optical biosensors, nanomaterials and composites for biosensing, wearable and physiological monitoring systems, bioelectronic sensors, and protein and nanomaterial engineering for biosensors.

Several papers in this Special Issue focus on molecular diagnostics and biomarker detection, including molecular detection systems, CRISPR-based assays for nucleic acid detection, and point-of-care viral biosensors and diagnostic platforms. These studies highlight the increasing importance of rapid, portable, and multiplexed diagnostic systems.

Another set of papers discusses wearable biosensors and physiological monitoring systems, including sensors for tracking lactate in sweat, tissue oxygenation, and arterial pulse strain, as well as ultrasound-based monitoring devices. These technologies highlight the growing importance of wearable, non-invasive biosensors for continuous monitoring of physiological parameters.

Nanomaterials and advanced materials for biosensing are prominently featured in this Special Issue, including nanomaterial-based sensing platforms such as surface-enhanced Raman spectroscopy (SERS) sensors, quantum dot-based detection methods, and carbon nanotube electrochemistry. These materials improve the sensitivity, selectivity, and overall performance of biosensors.

Bioelectronic sensing systems and the monitoring of biological systems are also discussed, including platforms for tracking neuronal network activity with bioelectronic measurement tools. These studies highlight the vital role of biosensors in researching biological systems and cellular networks.

Additionally, this Special Issue features review articles on protein, nucleic acid, and nanomaterial engineering for biosensors, emphasizing the importance of protein engineering, enzyme immobilization, and nanomaterials in the development of modern biosensing platforms.

The papers published in this Special Issue address many of these challenges by introducing new biosensor materials, improved sensing platforms, wearable monitoring systems, and molecular diagnostic tools, as well as review articles summarizing recent progress in biosensor engineering and analytical methods. Together, these contributions help reduce the gap between fundamental biosensor research and practical monitoring and diagnostic applications.

## 5. Future Perspectives

The future development of biosensors will likely be heavily impacted by advances in nanotechnology, synthetic biology, protein engineering, microfluidics, wearable electronics, and artificial intelligence. Combining biosensors with artificial intelligence and data analysis tools will enable the creation of smart diagnostic systems and personalized health monitoring.

Advances in protein engineering and synthetic biology will allow the development of new biological recognition elements with better stability, specificity, and catalytic efficiency. Innovative materials such as nanocomposites, metal–organic frameworks, and functional polymers will enable the development of more sensitive and durable biosensors.

Wearable and implantable biosensors will facilitate continuous monitoring of physiological parameters, which is vital for personalized medicine and preventive healthcare. Environmental monitoring and food safety will also remain key applications for biosensors. In the future, biosensors are expected to become fully integrated smart analytical systems capable of continuous monitoring, real-time data processing, and autonomous decision-making, which will greatly influence healthcare, environmental monitoring, and industrial biotechnology.

## 6. Conclusions

This Special Issue highlights recent advances in biosensor technologies and emphasizes the importance of interdisciplinary research that combines biotechnology, analytical chemistry, materials science, nanotechnology, electronics, and data science to develop modern biosensors for monitoring and diagnostic applications. We hope this Special Issue will contribute to further progress in biosensor technologies and inspire new research in this rapidly growing field.

